# Supplementary material for: Quantifying intra-tumoral genetic heterogeneity of glioblastoma toward precision medicine using MRI and a data-inclusive machine learning algorithm
Source: PLoS One. 2024 Apr 3;19(4):e0299267. doi: 10.1371/journal.pone.0299267 (PMC10990246; doi:10.1371/journal.pone.0299267)
Supplement: S1 Appendix — (DOCX) [file pone.0299267.s001.docx]

**Supporting Information**

**Proof of Proposition 1**

Let $\alpha_{i}^{(1)}$, $\alpha_{i}^{(2)},\beta_{j}^{\left( 12 \right)},\beta_{k}^{(3)},A_{i}^{(1)},A_{i}^{(2)},B_{j}^{(12)},B_{k}^{(3)},\mu\geq0$ be Lagrangian multipliers and $C_{1}$ and $C_{2}$ be tuning parameters. The Lagrangian for the primal WSO-SVM optimization in Eq. (1)-(7) is

$L=\frac{1}{2}w^{T}w+\sum_{i=1}^{n_{1}} \alpha_{i}^{(1)}\left( w^{T}\phi\left( x_{i}^{(1)} \right)-b_{1}+1-\xi_{i}^{(1)} \right)-$ $\sum_{i^{'}=1}^{n_{2}} \alpha_{i^{'}}^{(2)}\left( w^{T}\phi\left( x_{i^{'}}^{(2)} \right)-b_{1}-1+\xi_{i^{'}}^{(2)} \right)$

$+\sum_{k=1}^{m_{0}} \beta_{k}^{\left( 0 \right)}(w^{T}\phi\left( x_{k}^{\left( 0 \right)} \right)-b_{0}+1-\zeta_{k}^{(0)})-\sum_{j=1}^{m_{12}^{'}} \beta_{j}^{\left( 12 \right)}\left( w^{T}\phi\left( x_{j}^{(12)} \right)-b_{0}-1+\zeta_{j}^{\left( 12 \right)} \right) +C_{1}(\sum_{i=1}^{n_{1}} \xi_{i}^{(1)}+\sum_{i^{'}=1}^{n_{2}} \xi_{i^{'}}^{(2)})+C_{2}\left( \sum_{k=1}^{m_{0}} \zeta_{k}^{(0)}+\sum_{j=1}^{m_{12}^{'}} \zeta_{j}^{(12)} \right)-\sum_{i=1}^{n_{1}} A_{i}^{(1)}\xi_{i}^{(1)}-\sum_{i^{'}=1}^{n_{2}} A_{i^{'}}^{(2)}\xi_{i^{'}}^{(2)}-\sum_{k=1}^{m_{0}} B_{k}^{(0)}\zeta_{k}^{(0)}-\sum_{j=1}^{m_{12}^{'}} B_{j}^{(12)}\zeta_{j}^{(12)}+\mu(b_{0}-b_{1})$. (8)

Then the optimal solution of the primal problem in Eq. (1)-(7) is equivalent to the solution of the following optimization:

$\max_{\alpha,\beta,A,B,\mu} min_{w,b,\xi,\zeta}L$. (9)

The KKT conditions for the primal problem require the following to hold:

$\nabla_{w}L=w+\sum_{i=1}^{n_{1}} \alpha_{i}^{(1)}\phi\left( x_{i}^{(1)} \right)-\sum_{i^{'}=1}^{n_{2}} \alpha_{i^{'}}^{(2)}\phi\left( x_{i^{'}}^{(2)} \right)+ \sum_{k=1}^{m_{0}} \beta_{k}^{(0)}\phi\left( x_{k}^{(0)} \right)-\sum_{j=1}^{m_{12}^{'}} \beta_{j}^{(12)}\phi\left( x_{j}^{(12)} \right) =0$,

$\nabla_{b_{1}}L=-\sum_{i=1}^{n_{1}} \alpha_{i}^{\left( 1 \right)}+\sum_{i^{'}=1}^{n_{2}} \alpha_{i^{'}}^{\left( 2 \right)}-\mu=0$,

$\nabla_{b_{0}}L=-\sum_{k=1}^{m_{0}} \beta_{k}^{\left( 0 \right)}+\sum_{j=1}^{m_{12}^{'}} \beta_{j}^{\left( 12 \right)}+\mu=0$,

$\nabla_{\xi_{i}^{(1)}}L=-\alpha_{i}^{(1)}+C_{1}-A_{i}^{(1)}=0$, $i=1,...,n_{1}$,

$\nabla_{\xi_{i^{'}}^{(2)}}L=-\alpha_{i^{'}}^{(2)}+C_{1}-A_{i^{'}}^{(2)}=0$, $i^{'}=1,...,n_{2}$,

$\nabla_{\zeta_{k}^{(0)}}L=-\beta_{k}^{(0)}+C_{2}-B_{k}^{(0)}=0$, $k=1,...,m_{0}$.

$\nabla_{\zeta_{j}^{(12)}}L=-\beta_{j}^{(12)}+C_{2}-B_{j}^{(12)}=0$, $j=1,...,m_{12}^{'}$,

Then we have

$w=-\sum_{i=1}^{n_{1}} \alpha_{i}^{(1)}\phi\left( x_{i}^{(1)} \right)+\sum_{i^{'}=1}^{n_{2}} \alpha_{i^{'}}^{(2)}\phi\left( x_{i^{'}}^{(2)} \right)-\sum_{k=1}^{m_{0}} \beta_{k}^{(0)}\phi\left( x_{k}^{(0)} \right)+\sum_{j=1}^{m_{12}^{'}} \beta_{j}^{(12)}\phi\left( x_{j}^{(12)} \right)$, (10)

$\mu=-\sum_{i=1}^{n_{1}} \alpha_{i}^{\left( 1 \right)}+\sum_{i^{'}=1}^{n_{2}} \alpha_{i^{'}}^{(2)}$, (11)

$\mu=\sum_{k=1}^{m_{0}} \beta_{k}^{(0)}-\sum_{j=1}^{m_{12}^{'}} \beta_{j}^{(12)}$, (12)

$A_{i}^{(1)}=-\alpha_{i}^{(1)}+C_{1}$, $i=1,...,n_{1}$, (13)

$A_{i^{'}}^{(2)}=-\alpha_{i^{'}}^{(2)}+C_{1}$, $i^{'}=1,...,n_{2}$, (14)

$B_{k}^{(0)}=-\beta_{k}^{\left( 0 \right)}+C_{2}, k=1,...,m_{0},$ (15)

$B_{j}^{(12)}=-\beta_{j}^{(12)}+C_{2}$, $j=1,...,m_{12}^{'}$. (16)

Inserting Eq. (11)-(16) into the optimization in Eq. (9), after simplification we can get

$\max_{\alpha,\beta} L=\frac{1}{2}w^{T}w+\sum_{i=1}^{n_{1}} \alpha_{i}^{\left( 1 \right)}\left( w^{T}\phi\left( x_{i}^{\left( 1 \right)} \right)+1 \right)-\sum_{i^{'}=1}^{n_{2}} \alpha_{i^{'}}^{\left( 2 \right)}\left( w^{T}\phi\left( x_{i^{'}}^{\left( 2 \right)} \right)-1 \right)+\sum_{k=1}^{m_{0}} \beta_{k}^{\left( 0 \right)}\left( w^{T}\phi\left( x_{k}^{\left( 0 \right)} \right)+1 \right)-\sum_{j=1}^{m_{12}^{'}} \beta_{j}^{\left( 12 \right)}\left( w^{T}\phi\left( x_{j}^{\left( 12 \right)} \right)-1 \right)$. (17)

Furthermore, inserting Eq. (10) into the optimization in Eq. (17), we can have

$\max_{\alpha,\beta} L=-\frac{1}{2}\gamma^{T}YKY\gamma$+ $\sum_{i=1}^{n_{1}} \alpha_{i}^{(1)}+\sum_{i^{'}=1}^{n_{2}} \alpha_{i^{'}}^{2}+\sum_{k=1}^{m_{0}} \beta_{k}^{(0)}+\sum_{j=1}^{m_{12}^{'}} \beta_{j}^{(12)}$.

Additionally, the conditions in Eq. (11)-(12) give rise to the constraints of

$-\sum_{i=1}^{n_{1}} \alpha_{i}^{\left( 1 \right)}+\sum_{i^{'}=1}^{n_{2}} \alpha_{i^{'}}^{\left( 2 \right)}-\sum_{k=1}^{m_{0}} \beta_{k}^{(0)}+\sum_{j=1}^{m_{12}^{'}} \beta_{j}^{(12)}=0$,

$-\sum_{i=1}^{n_{1}} \alpha_{i}^{\left( 1 \right)}+\sum_{i^{'}=1}^{n_{2}} \alpha_{i^{'}}^{(2)}\geq0$.

The conditions in Eq. (13)-(16) give rise to the constraints of

$0\leq\alpha_{i}^{\left( 1 \right)}\leq C_{1}, i=1,...,n_{1};0\leq\alpha_{i^{'}}^{\left( 2 \right)}\leq C_{1}, i^{'}=1,...,n_{2}$,

$0\leq\beta_{k}^{\left( 0 \right)}\leq C_{2}, k=1,...,m_{0};0\leq\beta_{j}^{\left( 12 \right)}\leq C_{2}, j=1,...,m_{12}^{'}$.

Finally, the dual problem becomes

$\min_{\alpha,\beta} \frac{1}{2}\gamma^{T}YKY\gamma-\sum_{i=1}^{n_{1}} \alpha_{i}^{\left( 1 \right)}-\sum_{i^{'}=1}^{n_{2}} \alpha_{i^{'}}^{\left( 2 \right)}-\sum_{k=1}^{m_{0}} \beta_{k}^{(0)}-\sum_{j=1}^{m_{12}^{'}} \beta_{j}^{\left( 12 \right)}$,

subject to

$-\sum_{i=1}^{n_{1}} \alpha_{i}^{\left( 1 \right)}+\sum_{i^{'}=1}^{n_{2}} \alpha_{i^{'}}^{(2)}-\sum_{k=1}^{m_{0}} \beta_{k}^{(0)}+\sum_{j=1}^{m_{12}^{'}} \beta_{j}^{(12)}=0$,

$-\sum_{i=1}^{n_{1}} \alpha_{i}^{\left( 1 \right)}+\sum_{i^{'}=1}^{n_{2}} \alpha_{i^{'}}^{(2)}\geq0$,

$0\leq\alpha_{i}^{\left( 1 \right)}\leq C_{1}, i=1,...,n_{1};0\leq\alpha_{i^{'}}^{\left( 2 \right)}\leq C_{1}, i^{'}=1,...,n_{2}$,

$0\leq\beta_{k}^{\left( 0 \right)}\leq C_{2}, k=1,...,m_{0};0\leq\beta_{j}^{\left( 12 \right)}\leq C_{2}, j=1,...,m_{12}^{'}$. ▄

**MRI protocols, parametric maps, and image co-registration**

The MRI images used in this study were obtained through standard protocols and gone through preprocessing steps for quality control, which were described in detail in our previous publications [1]–[3]. Here we provide an exertion of the detailed approaches from a prior paper [1].

We performed all imaging at 3 T field strength (Sigma HDx; GE-Healthcare Waukesha Milwaukee; Ingenia, Philips Healthcare, Best, Netherlands; Magnetome Skyra; Siemens Healthcare, Erlangen Germany) within 1 day prior to stereotactic surgery. Conventional MRI included standard pre- and post-contrast T1-Weighted (T1-C, T1+C, respectively) and pre-contrast T2-Weighted (T2W) sequences. T1W images were acquired using spoiled gradient recalled-echo inversion-recovery prepped (SPGR-IR prepped) (TI/TR/TE = 300/6.8/2.8 ms; matrix = 320 × 224; FOV = 26 cm; thickness = 2 mm). T2W images were acquired using fast-spin-echo (FSE) (TR/TE = 5133/78 ms; matrix = 320 × 192; FOV = 26 cm; thickness = 2 mm). T1 + C images were acquired after completion of Dynamic Susceptibility-weighted Contrast-enhanced (DSC) Perfusion MRI (pMRI) following total Gd-DTPA (gadobenate dimeglumine) dosage of 0.15 mmol/kg as described below [2], [4], [5]. Diffusion Tensor (DTI): DTI imaging was performed using Spin-Echo Echo-planar imaging (EPI) [TR/TE 10,000/85.2 ms, matrix 256 × 256; FOV 30 cm, 3 mm slice, 30 directions, ASSET, B = 0,1000]. The original DTI image DICOM files were converted to a FSL recognized NIfTI file format, using MRIConvert (<http://lcni.uoregon.edu/downloads/mriconvert>), before processing in FSL from semi-automated script. DTI parametric maps were calculated using FSL (<http://fsl.fmrib.ox.ac.uk/fsl/fslwiki/>), to generate whole-brain maps of mean diffusivity (MD) and fractional anisotrophy (FA) based on previously published methods [6]. DSC-pMRI: prior to DSC acquisition, preload dose (PLD) of 0.1 mmol/kg was administered to minimize T1W leakage errors. After PLD, we employed Gradient-echo (GE) EPI [TR/TE/flip angle = 1500 ms/20 ms/60°, matrix 128 × 128, thickness 5 mm] for 3 min. At 45 s after the start of the DSC sequence, we administered another 0.05 mmol/kg i.v. bolus Gd-DTPA [2], [4], [5]. The initial source volume of images from the GE-EPI scan contained negative contrast enhancement (i.e., susceptibility effects from the PLD administration) and provided the MRI contrast labeled EPI+C. At approximately 6 min after the time of contrast injection, the T2*W signal loss on EPI+C provides information about tissue cell density from contrast distribution within the extravascular, extracellular space [2], [7]. We performed leakage correction and calculated relative cerebral blood (rCBV) based on the entire DSC acquisition using IB Neuro (Imaging Biometrics, LLC) as referenced [8], [9]. We also normalized rCBV values to contralateral normal appearing white matter as previously described [2], [5]. Image coregistration: for image coregistration, we employed tools from ITK ([www.itk.org](http://www.itk.org/)) and IB Suite (Imaging Biometrics, LLC) as previously described [2], [4], [5]. All datasets were coregistered to the relatively high quality DTI B0 anatomical image volume. This offered the additional advantage of minimizing potential distortion errors (from data resampling) that could preferentially impact the mathematically sensitive DTI metrics. Ultimately, the coregistered data exhibited in plane voxel resolution of ~ 1.17 mm (256 × 256 matrix) and slice thickness of 3 mm.

**Feature extracted from regional MRI**

The MRI features corresponding to each biopsy sample were extracted from a defined “region”, i.e., an 8x8 pixel^2^ window centered at the sampling location. From this window, we extracted 56 features from each of the five MRI contrast images, which included 18 statistical features and 26 and 12 texture features using two well-established texture analysis algorithms, Gray-Level Co-occurrence Matrix (GLCM) and Gabor Filters (GF), respectively. The statistical features include commonly used ones in the literature [10], such as mean and standard deviation of gray-level intensities, Energy, Total Energy, Entropy, Minimum, 10th percentile, 90th percentile, Maximum, Median, Interquartile Range, Range, Mean Absolute Deviation (MAD), Robust Mean Absolute Deviation (rMAD), Root Mean Squared (RMS), Skewness, Kurtosis, Uniformity. Before applying GLCM and GF, we mapped the intensity values within the window onto the range of 0–255. This step helped standardize intensities and reduced effects of intensity nonuniformity on features extracted during subsequent texture analysis. The GLCM algorithm produced 26 features by setting the distance parameter to be 1 and 3 to capture different scales of spatial patterns [11], [12], such as Angular Second Moment Average, Contrast Average, Correlation Average, Sum of Squares Variance Average, Inverse Difference Moment Average, Average of Sum Average, Average of Sum Variance, Average of Sum Entropy, Entropy Average, Average of Difference Variance, Average of Difference Entropy, Average of Information Measure of Correlation 1, Average of Information Measure of Correlation 2. The GF algorithm produces 12 features, including Gabor Mean and Gabor Standard Deviation, by setting sigma to be 0.4, 0.7 and frequency to be 0.1, 0.3, 0.5 to capture different frequency and orientation contents [13].

**References**

[1] L. S. Hu *et al.*, “Uncertainty quantification in the radiogenomics modeling of EGFR amplification in glioblastoma,” *Sci Rep*, vol. 11, no. 1, p. 3932, Feb. 2021, doi: 10.1038/s41598-021-83141-z.

[2] L. S. Hu *et al.*, “Multi-Parametric MRI and Texture Analysis to Visualize Spatial Histologic Heterogeneity and Tumor Extent in Glioblastoma,” *PLoS One*, vol. 10, no. 11, p. e0141506, Nov. 2015, doi: 10.1371/journal.pone.0141506.

[3] N. Gaw *et al.*, “Integration of machine learning and mechanistic models accurately predicts variation in cell density of glioblastoma using multiparametric MRI,” *Sci Rep*, vol. 9, no. 1, p. 10063, Jul. 2019, doi: 10.1038/s41598-019-46296-4.

[4] L. S. Hu *et al.*, “Radiogenomics to characterize regional genetic heterogeneity in glioblastoma,” *Neuro Oncol*, vol. 19, no. 1, pp. 128–137, Jan. 2017, doi: 10.1093/neuonc/now135.

[5] L. S. Hu *et al.*, “Reevaluating the imaging definition of tumor progression: perfusion MRI quantifies recurrent glioblastoma tumor fraction, pseudoprogression, and radiation necrosis to predict survival,” *Neuro Oncol*, vol. 14, no. 7, pp. 919–930, Jul. 2012, doi: 10.1093/neuonc/nos112.

[6] S. J. Price *et al.*, “Improved delineation of glioma margins and regions of infiltration with the use of diffusion tensor imaging: an image-guided biopsy study.,” *AJNR Am J Neuroradiol*, vol. 27, no. 9, pp. 1969–74, Oct. 2006.

[7] N. B. Semmineh *et al.*, “Assessing tumor cytoarchitecture using multiecho DSC-MRI derived measures of the transverse relaxivity at tracer equilibrium (TRATE).,” *Magn Reson Med*, vol. 74, no. 3, pp. 772–84, Sep. 2015, doi: 10.1002/mrm.25435.

[8] J. L. Boxerman, K. M. Schmainda, and R. M. Weisskoff, “Relative cerebral blood volume maps corrected for contrast agent extravasation significantly correlate with glioma tumor grade, whereas uncorrected maps do not.,” *AJNR Am J Neuroradiol*, vol. 27, no. 4, pp. 859–67, Apr. 2006.

[9] L. S. Hu *et al.*, “Impact of Software Modeling on the Accuracy of Perfusion MRI in Glioma.,” *AJNR Am J Neuroradiol*, vol. 36, no. 12, pp. 2242–9, Dec. 2015, doi: 10.3174/ajnr.A4451.

[10] A. Zwanenburg, S. Leger, M. Vallières, and S. Löck, “Image biomarker standardisation initiative,” Dec. 2016, doi: 10.1148/radiol.2020191145.

[11] T. Ojala, M. Pietikainen, and T. Maenpaa, “Multiresolution gray-scale and rotation invariant texture classification with local binary patterns,” *IEEE Trans Pattern Anal Mach Intell*, vol. 24, no. 7, pp. 971–987, Jul. 2002, doi: 10.1109/TPAMI.2002.1017623.

[12] R. M. Haralick, K. Shanmugam, and I. Dinstein, “Textural Features for Image Classification,” *IEEE Trans Syst Man Cybern*, vol. SMC-3, no. 6, pp. 610–621, Nov. 1973, doi: 10.1109/TSMC.1973.4309314.

[13] A. G. Ramakrishnan, S. Kumar Raja, and H. V. Raghu Ram, “Neural network-based segmentation of textures using Gabor features,” in *Proceedings of the 12th IEEE Workshop on Neural Networks for Signal Processing*, IEEE, pp. 365–374. doi: 10.1109/NNSP.2002.1030048.
