# Supplementary figures and images for: Quantifying intra-tumoral genetic heterogeneity of glioblastoma toward precision medicine using MRI and a data-inclusive machine learning algorithm
Source: PLoS One. 2024 Apr 3;19(4):e0299267. doi: 10.1371/journal.pone.0299267 (PMC10990246; doi:10.1371/journal.pone.0299267)

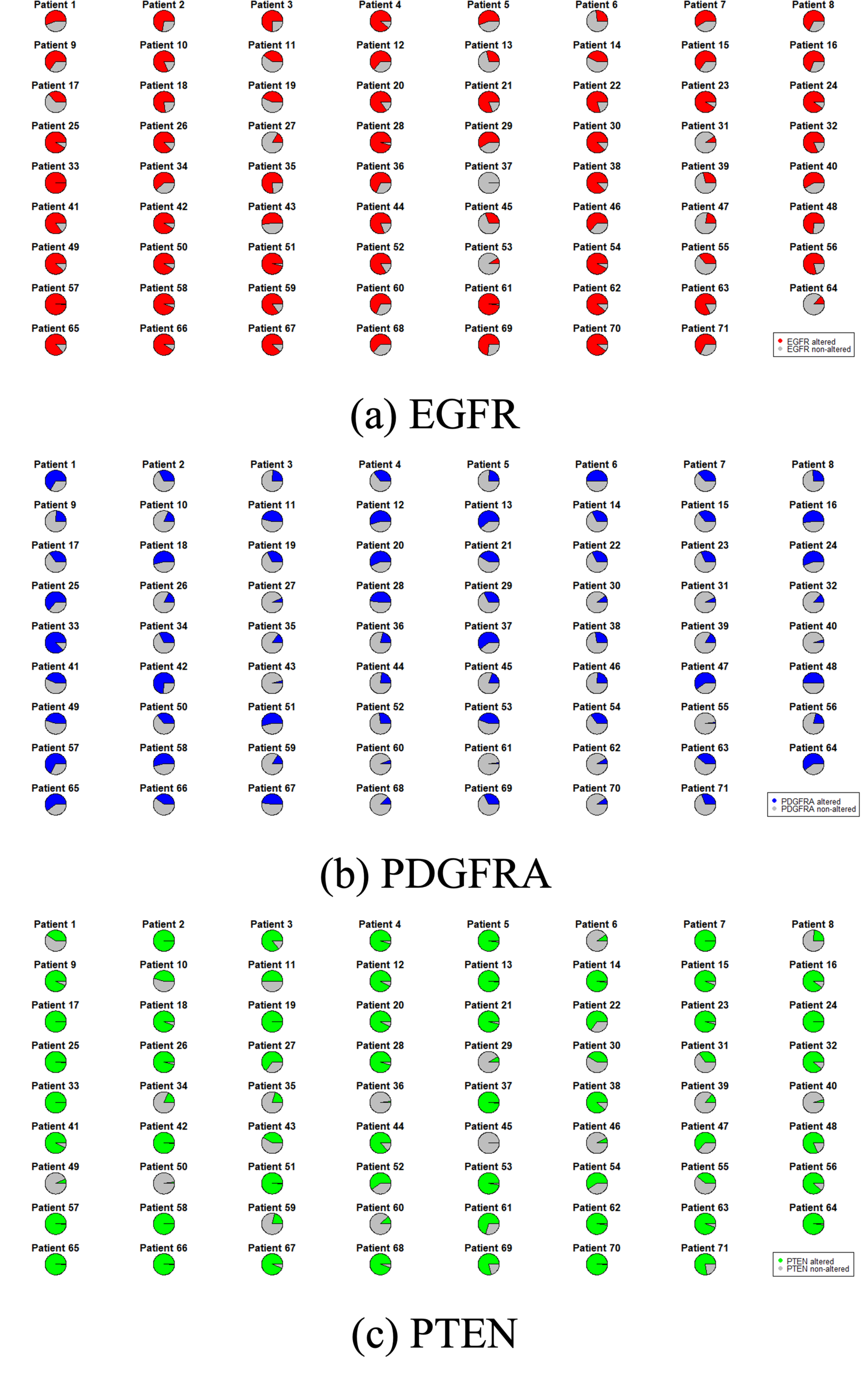

Supplement: S1 Fig — Patient-wise proportions of alteration vs. non-alteration for (a) EGFR, (b) PDGFRA, and (c) PTEN within tumoral AOI, aggregated from the prediction maps of these genes by WSO-SVM. (TIF) [file pone.0299267.s002.tif]
